# Lung-homing nanoliposomes for early intervention in NETosis and inflammation during acute lung injury

**DOI:** 10.1186/s40580-025-00475-4

**Published:** 2025-02-03

**Authors:** Jungbum Kim, Donghyuk Seo, So-Yeol Yoo, Hye-Jin Lee, Jisun Kim, Ji Eun Yeom, Jae-Young Lee, Wooram Park, Kyung Soo Hong, Wonhwa Lee

**Affiliations:** 1 Department of Chemistry, Sungkyunkwan University, Suwon, 16419 Republic of Korea; 2 College of Pharmacy and Research Institute of Pharmaceutical Sciences, Seoul National University, Seoul, 08826 Republic of Korea; 3 Department of Integrative Biotechnology, College of Biotechnology and Bioengineering, Sungkyunkwan University, Suwon, Gyeonggi 16419 Republic of Korea; 4 Department of MetaBioHealth, Institute for Cross-disciplinary Studies, Sungkyunkwan University, Suwon, Gyeonggi 16419 Republic of Korea; 5 Division of Pulmonology and Allergy, Department of Internal Medicine, College of Medicine, Regional Center for Respiratory Diseases, Yeungnam University, Yeungnam University Medical Center, Daegu, 42415 Republic of Korea

**Keywords:** Lung-homing nanoliposome, NETosis, Acute respiratory distress syndrome (ARDS), DNase-1, Sivelestat

## Abstract

Acute lung injury (ALI) is characterized by severe inflammation in lung tissue, excessive immune response and impaired lung function. In hospitalized high-risk patients and cases of secondary infection due to surgical contamination, it can lead to higher mortality rates and require immediate intervention. Currently, clinical treatments are limited in symptomatic therapy as mechanical ventilation and corticosteroids, having insufficient efficacy in mitigating the cause of progression to severe illness. Here we report a pulmonary targeting lung-homing nanoliposome (LHN) designed to attenuate excessive Neutrophil Extracellular Trap formation (NETosis) through sivelestat and DNase-1, coupled with an anti-inflammatory effect mediated by 25-hydroxycholesterol (25-HC), offering a promising intervention for the acute phase of ALI. Through intratracheal delivery, we intend prompt and constant action within the lungs to effectively prevent excessive NETosis. Isolated neutrophils from blood samples of severe ARDS patients demonstrated significant anti-NETosis effects, as well as reduced proinflammatory cytokine secretion. Furthermore, in a murine model of LPS-induced ALI, we confirmed improvements in lung histopathology, and early respiratory function. Also, attenuation of systemic inflammatory response syndrome (SIRS), with notable reductions in NETosis and neutrophil trafficking was investigated. This presents a targeted therapeutic approach that can be applied in early stages of high-risk patients to prevent severe pulmonary disease progression.

**Supplementary Information:**

The online version contains supplementary material available at 10.1186/s40580-025-00475-4.

## Graphical Abstract

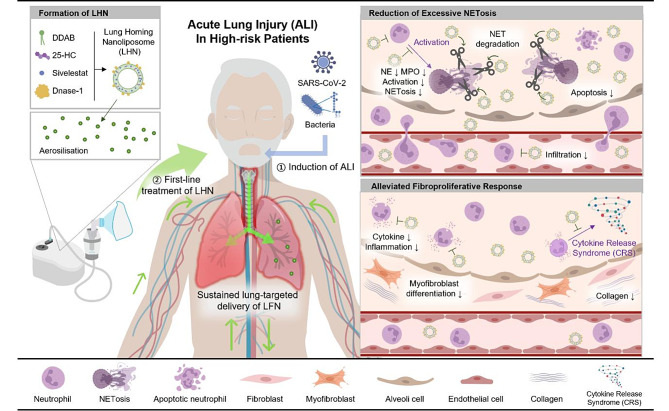

## Introduction

Acute Respiratory Distress Syndrome (ARDS) represents a significant global health challenge due to its association with severe inflammatory lung damage that can lead to type I respiratory failure. This condition is frequently exacerbated by cytokine release syndrome (CRS), which accelerates lung tissue injury, promotes fibrosis, and may precipitate multiple organ failure (MOF) [1–3]. The systemic inflammatory response associated with ARDS often extends beyond the lungs, compromising the function of vital organs such as the kidneys, liver, and cardiovascular system. While current ARDS management relies heavily on supportive care, such as mechanical ventilation and oxygen therapy, existing pharmacological options such as corticosteroids remain limited in their efficacy to reduce inflammation and prevent ongoing pulmonary damage [4, 5]. In some cases, mechanical ventilation itself can exacerbate ARDS by causing additional lung injury, a phenomenon known as ventilator-induced lung injury (VILI) [6, 7]. When caused by infection, misuse and aggressive dosing of various antibiotics in response can lead to severe side effects and promote antibiotic resistance [8, 9]. Secondary infection following surgical contamination or antibiotics resistance can progress to a more severe stage of ARDS, necessitating immediate intervention. This underscores the limitations of current treatment approaches and highlights the need for targeted pharmacological interventions that can address the underlying inflammatory processes without contributing to further lung damage.

Amid these circumstances, the COVID-19 pandemic provided an opportunity to gather a diverse and extensive patient database, along with a wealth of associated research findings. In a study across 29,144 patients admitted to ICUs in 50 countries, 10.4% met the criteria for ARDS, with 23.4% of these cases classified as severe ARDS with a hospital mortality rate of 46.1% (95% CI, 41.9-50.4%) [10]. In studies focused on COVID-19-associated ARDS, COVID-19 patients transferred to an ICU, nearly 2/3 (63%) receive IMV and 3/4 (75%) have ARDS. The mortality rate of ICU COVID-19 patients is 40% and of those who receive IMV 59%; the mortality rate in COVID-19-associated ARDS is 45%, and the incidence of ARDS among non-survivors of COVID-19 is 90% [11].

Neutrophils play a pivotal role in the pathogenesis of ARDS, particularly during the exudative and proliferative phases [12, 13]. In the exudative phase, neutrophils infiltrate pulmonary tissue and release neutrophil extracellular traps (NETs) as a defense mechanism against pathogens. These web-like structures, consisting of DNA strands, myeloperoxidase (MPO), neutrophil elastase (NE), and citrullinated histones (cit-H3), can become detrimental when excessively produced, exacerbating the progression of ARDS to a severe state. NE progresses chromatin decondensation during NET formation, degrades extracellular matrix components, further compromising barrier integrity. Cit-H3 amplifies inflammatory responses by promoting the recruitment and activation of immune cells through its immunogenic properties. cfDNA acts as a potent damage-associated molecular pattern (DAMP) that amplifies inflammation by activating pattern recognition receptors (PRRs) such as TLR9. Concurrently, MPO, another key NET component, contributes to oxidative stress by generating reactive oxygen species (ROS), further intensifying tissue damage.

As ARDS progresses into the proliferative phase, the sustained presence of NETs perpetuates inflammation and promotes fibroproliferation, further impairing lung function and setting the foundation for fibrosis [14–16]. Thus, targeted modulation of neutrophils and NETs during both the exudative and proliferative stages is, therefore, imperative to mitigate disease severity and enhance clinical outcomes. Such approach underscores the need for precise therapeutic strategies that go beyond simple suppression of inflammation, directly addressing neutrophil-mediated damage to prevent fibrosis and promote patient recovery [17–20].

In response to these demands, research aimed at inhibiting NETosis has gained significant attention, with some potential anti-NETosis therapeutics in clinical/pre-clinical study. These drugs, such as disulfiram, BMS-P5, AZD9668, can effectively block NETosis related diseases as type I respiratory failures through various mechanisms [21]. However, their efficacy is limited due to short half-lives, indirect inhibition of NET production, as well as non-specific targeting. Sivelestat and DNase-1, each address distinct aspects of neutrophil-mediated damage. Sivelestat, a small-molecule neutrophil elastase (NE) inhibitor, is approved in Japan and South Korea for ARDS associated with systemic inflammatory response syndrome (SIRS) and has demonstrated clinical efficacy. Clinically, in a Phase III trial with 230 patients with ALI induced by SIRS, sivelestat demonstrated a positive impact by improving lung function, reducing the duration of mechanical ventilation, and shortening ICU stays [22]. DNase-1 degrades the DNA backbone of NETs, thereby reducing their harmful accumulation and alleviating lung tissue damage [23, 24]. Together, sivelestat and DNase-1 provide targeted reduction of NETosis and inflammation, effectively mitigating the detrimental impact of neutrophils on lung tissue and reducing the risk of systemic inflammation.

In this study, we developed an innovative lung homing nanoliposome (LHN) that incorporates both sivelestat and DNase-1 into a lung-targeted delivery system. This nanoparticle construct, consisting of 25-hydroxycholesterol (25-HC) conjugated with didodecyldimethylammonium bromide (DDAB), is specifically designed to target and concentrate within lung tissue, facilitating localized and efficient delivery of therapeutic agents. Additionally, 25-HC in known to modulate NF-κB and SREBP2 pathways, which are essential in driving inflammation and maintaining cholesterol homeostasis [25]. This multi-modal approach facilitates an overall reduction in inflammation, while also curtailing fibroproliferation and protecting lung tissue from ongoing damage. Importantly, the lung-targeted nature of the LHN minimizes off-target effects and increases half-life, ensuring therapeutic agents are precisely in action where needed.

## Materials and methods

### Materials

DNase-I (cat no. 11284932001) was purchased from Roche (Basel, Switzerland). Didodecyldimethylammonium bromide, 99% (cat no. 407120250) and UltraPure™ Salmon Sperm DNA Solution (cat no. 15632011) were purchased from Thermo Scientific™. 25-Hydroxycholesterol (cat no. H1015) was obtained from Sigma-Aldrich (PA, USA). Percoll™ (Density 1.130 g/mL, cat no. 17089101) was purchased from Cytiva. Cy5.5 amine was purchased from BioActs (Incheon, Korea).

### Animal and husbandry

5-week-old male ICR mice were obtained from RaonBio (Yongin, Korea) and used after a one-week acclimatization period. The mice were housed in groups of three per cage under controlled environmental conditions: temperature maintained at 20–25 °C, humidity at 40–45%, and a 12-hour light/dark cycle. They were provided with a standard rodent pellet diet (Envigo) and had access to water ad libitum. All experimental procedures were performed in compliance with the guidelines of the Institutional Animal Care and Use Committee (IACUC) of Sungkyunkwan University (IACUC Approval No.: 202111291).

### Animal model

To establish LPS-induced ALI mouse model, 6-week-old male ICR mice were anesthetized with isoflurane and administered 10 mg/kg of lipopolysaccharide (LPS) in 20 µL of sterile PBS via intratracheal instillation using a microsprayer (BioJane). After LPS administration, the mice were returned to their cages and monitored for signs of respiratory distress. One hour later, sivelestat and DNase-1 or LHN were administered via intratracheal administration. At the endpoint (13 h post-administration), the mice were euthanized, and samples such as bronchoalveolar lavage fluid (BALF), lung tissue or plasma were collected.

### Plasma sample

Whole blood was collected from patients admitted at Yeungnam University Medical Center after diagnosis with SARS-CoV-2 infection at a public health center in Daegu, Republic of Korea. Patients with COVID-19 sepsis were defined using criteria provided by the Sepsis Consensus Conference Committee.

Eligible patients met the following criteria: age, over 18 years; and an acute onset medical condition with at least one of the following criteria: fever (tympanic temperature ≥ 38 °C at the nurse triage), suspected systemic infection, two or more systemic inflammatory response syndrome SIRS criteria, hypotension (systolic blood pressure < 90 mmHg), and/or shock. Healthy volunteers were used as controls. Clinical data were collected for all the patients. Plasma samples were prepared by centrifugation at 2000×g for 5 min within 12 h after whole blood collection. The human study protocol was approved by the Institutional Review Board of Yeungnam University Hospital at Daegu in Korea (YUH 2020-03-057 and 2020-05-031-001).

### Preparation and characterization of LHN

LHN was prepared using a self-assembly process via the thin-film dispersion method. Briefly, DDAB (4 mg) was dissolved in methanol (200 µL), while 25-hydroxycholesterol (1 mg) and sivelestat were dissolved in ethanol (100 µL, 200 µL). The solutions were combined and evaporated using a nitrogen evaporator (MG-2200; Eyela, Tokyo, Japan) at 60˚C to form a lipid film. Ultrapure water (900 µL), purified using both a RIOs 100 and a US/Super-Q^®^ Plus water purification system (Millipore, Burlington, MA, USA), was added to the lipid film, followed by ultrasonication using an ultrasonic processor (VC-505; Sonics & Materials, Inc., Newtown, CT, USA) at 20% amplitude with a pulsing cycle of 2 s on and 3 s off for 1.5 min. The solution was filtered through 0.45-µm syringe filter (Minisart RC 15; Sartorius, Göttingen, Germany) to remove precipitated (unloaded) sivelestat. Then DNase-1 (1 mg) was dissolved in PBS (100 µL), added to the filtrate, and subjected to ultrasonication under the same conditions.

The mean particle diameter, polydispersity index, and zeta potential of LHN (before and after addition of DNase-1) were measured using dynamic light scattering (DLS) and laser Doppler electrophoresis techniques, respectively, with a Zetasizer Ultra (Malvern Instruments Ltd., Malvern, UK). The morphology of the nanoliposomes was assessed using transmission electron microscopy (TEM; Talos L120C; FEI, Hillsboro, OR, USA) at a cryogenic temperature. The encapsulation efficiency (EE) of sivelestat in LHN was determined using high-performance liquid chromatography (HPLC) on a Shimadzu Nexera XR Modular HPLC system (Shimadzu, Kyoto, Japan) equipped with a reverse-phase Kinetex C18 column (4.6 × 100 mm, 2.6 μm; Phenomenex, Torrance, CA, USA) and a guard column (C18, 4 × 2.0 mm; Phenomenex). LHN was disrupted with acetonitrile (ACN) (dilution factor: 100) for analysis. The mobile phase consisted of ACN and deionized distilled water (90:10, v/v), with a flow rate of 1.0 mL/min and an injection volume of 20 µL. Detection was performed at 250 nm, and the retention time and total run time were 1.1 min and 3.0 min, respectively. EE was calculated according to the equation:

EE (%) = (Actual amount of sivelestat in LHN) / (Theoretical amount of sivelestat in LHN) × 100.

### DNA degradation assay

The DNase-1 activity was assessed using DNA degradation assay, with salmon sperm DNA (Thermo Fisher Scientific) as the cleavage substrate. The assay was performed using either free DNase-1 or LHN in 20 µL TAE(Tris-acetate-EDTA) buffer containing 2 µg of salmon sperm DNA. The reaction was incubated at 37 °C for 10 min, followed by addition of 1 unit of free DNase-1, by varying amounts of 5 mg/mL LHN (0.1–50 µL), or 10 µL of uncoated control nanoparticles (25-HC@DDAB-sivelestat without immobilized DNase-1). After incubation, the samples were subjected to electrophoresis on a 1% agarose gel and stained with SYBR safe DNA gel stain (Invitrogen) for 35 min. DNA degradation was visualized using the LuminoGraph II (ATTO) system, equipped with standard excitation and emission filters.

### Immunohistochemistry

Mice from each group were administered an intratracheal injection of LPS at a dose of 10 mg/kg. 13 h after injection lung tissues were carefully harvested after euthanasia. Lung tissue Sect. (10 μm) were fixed in 4% paraformaldehyde solution (Biosesang) prepared in PBS at pH 7.4. Permeabilization was performed using 0.05% Triton X-100 in PBS for 10 min. Subsequently, the slides were incubated overnight at 4 °C with either rabbit anti-collagen I antibody (ab34710, Abcam) or mouse anti-α-smooth muscle actin antibody (ab7817, Abcam). The following day, slides were incubated with secondary antibodies—anti-rabbit Alexa Fluor 488 (A11034, Invitrogen) or anti-mouse Alexa Fluor 594 (A11032, Invitrogen)—for 1 h at room temperature. After washing with PBS-T, the slides were mounted using fluorescence mounting medium (Dako) and analyzed using a fluorescence microscope (Leica Microsystems, Germany).

### TUNEL assay

Cryopreserved lung tissues were sectioned at 10 μm thickness and fixed in 4% paraformaldehyde (Biosesang) in PBS (pH 7.4) for 20 min at room temperature. After washing with PBS, tissue sections were permeabilized with 0.1% Triton X-100 in PBS for 2 min on ice and washed twice with PBS. The sections were then incubated with the TUNEL reaction mixture (11684795910, Roche) for 1 h at 37 °C in a humidified chamber. Following incubation, the sections were washed with PBS and mounted with fluorescence mounting medium (Dako). TUNEL-positive cells were visualized using a fluorescence microscope (Leica Microsystem).

### Neutrophil isolation from severe COVID-19 patient blood

Peripheral blood mononuclear cells (PBMCs) were isolated from whole blood collected from COVID-19 patients. Blood samples were diluted 1:1 with PBS and carefully layered onto Ficoll-Paque PLUS (GE Healthcare) in a 15 mL conical tube. The tubes were centrifuged at 400 g for 30 min at room temperature without brake. After centrifugation, the PBMC layer, located at the plasma-Ficoll interface, was carefully collected and washed twice with PBS at 100 g for 10 min. The collected PBMCs were resuspended in RPMI-1640 medium. A discontinuous Percoll gradient was prepared by layering 62%, 82% and 90% Percoll (GE Healthcare) solutions in a tube. The PBMC suspension was then added on top of the Percoll gradient and centrifuged at 2,500 rpm for 20 min at 4 °C. Neutrophils were enriched at the 62%/82% interface, collected, and washed with PBS to remove residual Percoll. To further purify the neutrophil population, the cell suspension was incubated with anti-CD45 antibody-conjugated magnetic beads (Miltenyi Biotec). The labeled cells were then passed through a MACS separation column in a magnetic field, allowing CD45 + neutrophils to be retained in the column while other cell types were washed away. The retained neutrophils were eluted from the column and washed with PBS. Viability of the neutrophils was generally > 95% as assessed by trypan blue dye exclusion.

### MPO ELISA

To quantify the release of granule matrix proteins upon degranulation, plasma from COVID-19-infected patients and both plasma and BALF from an ALI mouse model were analyzed. For human plasma samples, a human MPO ELISA kit (BMS2038INST, Invitrogen) was used, while both plasma and BALF from the mouse model were analyzed using a mouse myeloperoxidase ELISA kit (MBS700747, MyBioSource).

### NET ELISA

Freshly Isolated neutrophils were resuspended in RPMI-1640 medium and seeded in plates (1 × 10^5^ cells). Cells were stimulated with 25 nM phorbol-myristate acetate (PMA, Sigma-Aldrich) to induce NET formation. A treatment group was incubated with the sivelestat and DNase-1 or LHL along with PMA. After incubation, the presence of extracellular DNA (a marker of NETs) was measured using a fluorometric assay. To quantify NET formation, 1 µM of SYTOX Green (Invitrogen) was added to the wells. Fluorescence intensity was measured using a microplate reader with excitation at 488 nm and emission at 520 nm. The fluorescence signal correlates with the amount of DNA released, reflecting NET production. NET production was measured as arbitrary fluorescent units (AFUs).

### Cit-histone H3 ELISA

Cit-histone H3 concentrations in plasma or BALF from ALI mice model were determined using a human citrullinated histone H3 ELISA Kit (MBS7254090, MyBioSource).

### Quantification of plasma cfDNA

For isolation of cfDNA, plasma or BALF from COVID-19-infected patients and ALI mice models were processed. A Qiagen QIAamp DNA Mini Blood Kit (Qiagen) was used according to the manufacturer’s instructions, following centrifugation of the samples at 16,800 g for 10 min. Purified cfDNA was quantified using NANO-300 Micro Spectrophotometer (ALLSHENG).

### ELISA for DNase-1 activity

Serum and BALF samples were diluted 1:50 with digestion buffer spiked with 1 µg/mL of double stranded DNA. Samples were stained with PicoGreen (Invitrogen) according to the manufacturer’s instruction and incubated at 37 °C for 5 h. The reduction in PicoGreen staining (fluorescence emission, Em) was then measured using a fluorometer.

### Cytokine profiling

Cytokine levels in neutrophils derived from patients and in mouse samples (BALF and plasma) treated with either sivelestat and DNase-1 or LHN were analyzed using the Human XL Cytokine Array Kit (R&D Systems) according to the manufacturer’s instructions. The developed films were scanned, and the images were analyzed using ImageJ version 1.43 to quantify cytokine expression across the treatment groups.

### Hematoxylin and eosin staining

To induce ALI, male ICR mice were administered 10 mg/kg of LPS via intratracheal injection. 1-hour post-LPS administration, the mice received either PBS, sivelestat and DNase-1, or LHN via intratracheal instillation. 13 h after LPS injection, the mice were euthanized, and lung tissues were harvested and fixed in 4% paraformaldehyde (Biosesang). Following fixation, the lungs were dehydrated in a sucrose-PBS solution and embedded in Optimal cutting temperature compound (Sakura). Cryopreserved lung sections were prepared at a thickness of 10 μm and stained with hematoxylin and eosin (H&E). H&E staining was performed to evaluate the structural integrity of the lung and airway architecture, assess tissue edema and visualize the infiltration of inflammatory cells into the lung parenchyma. This analysis enabled the assessment of pathological changes in response to treatments and the extent of lung injury, including structural disruption and inflammatory cell infiltration.

### Lung function assessment

Lung function parameters were assessed using the flexiVent system (SCIREQ Scientific Respiratory Equipment, Montréal, Québec, Canada), which employs the forced oscillation technique (FOT) to measure respiratory mechanics. Mice were anesthetized with avertin (250 mg/kg, intraperitoneal injection) and subsequently administered pancuronium bromide (0.8 mg/kg, intraperitoneal) to suppress spontaneous respiratory efforts.

After the trachea was isolated, tracheostomy was performed using an 18-gauge, 30 mm cannulae. Then mice were connected to the flexiVent FX system and mechanically ventilated. Various pressure patterns were applied using FOT to measure lung volumes, including tidal volume (TV), inspiratory reserve volume (IRV), expiratory reserve volume (ERV), residual volume (RV), functional residual capacity (FRC), vital capacity (VC), and total lung capacity (TLC). Each measurement was repeated three times per mouse. Data were recorded and analyzed using the flexiVent operating software (version 8.3.0.4730). Respiratory parameters such as resistance (R), elastance (E), and compliance (C) were calculated. Pressure-volume (P–V) curves were generated to assess quasi-static compliance and tissue elasticity.

### Hydroxyproline and collagen assay

Mice were administered an intratracheal injection of LPS (10 mg/mL). After 13 h, the mice were euthanized, and lung tissue and BALF were collected for further analysis. Hydroxyproline concentration was measured in lung tissue using the PicoSens™ Hydroxyproline Assay Kit (BIOMAX, BM-HYP-100), and collagen concentration was assessed in BALF using the Sircol Collagen Assay Kit (Biocolor Ltd., Newtownabbey, UK). Both assays were performed according to the manufacturer’s protocols. Absorbance values were recorded at 560 nm using an ELISA plate reader (Tecan, Männedorf, Switzerland).

### In vivo biodistribution of Cy5.5-labeled LHN

To label LHN with fluorescence, Cy5.5 was added to the solution before the evaporation process of lipid film formation as previously described [25]. Cy5.5-labeled LHN were dispersed in 1 mL of PBS, volume of 40 µl was injected intratracheally per mouse. Real-time in vivo fluorescence imaging of the LHN-treated mice was performed at 0, 3, 6, 9, 12, and 24 h post-administration. At each time point, animals were euthanized, and major organs (heart, lung, liver, kidneys, and spleen) were harvested for ex vivo imaging and quantification of fluorescence intensity. For LPS-challenged mice receiving Cy5.5-labeled LHN 1 h post-LPS administration, in vivo fluorescence imaging was conducted at 0, 3, 6, and 12 h, with ex vivo fluorescence intensity analyzed at 12 and 24 h.

### Cytokine levels in serum and BALF of ALI mice

Fresh serum and BALF were used for analysis of IL-1β and IL-6 levels using mouse ELISA kits (Quantikine ELISA, R&D Systems, Minneapolis, MN, USA) according to the manufacturer’s instructions. The results were expressed as pg/mL.

### NET staining of lung tissue

Mice from each group were administered an intratracheal injection of LPS at a dose of 10 mg/kg. Thirteen hours after injection, the mice were euthanized, and their lung tissues were carefully harvested for further analysis. Lung tissue Sect. (10 μm) were fixed in 4% paraformaldehyde solution (Biosesang) prepared in PBS at pH 7.4 and washed twice with PBS. After washing the slides, 1 µM SYTOX Orange in PBS was applied to the tissue sections, followed by incubation at 4 °C for 30 min. After incubation, the slides were mounted using fluorescence mounting medium (Dako) and analyzed using a fluorescence microscope (Leica Microsystems, Germany).

### Clinical chemistry and cell counting in mouse whole blood and BALF

Fresh serum was used to assay alanine aminotransferase (ALT), aspartate aminotransferase (AST), blood urea nitrogen (BUN), creatinine (CRE), albumin (ALB) and lactate dehydrogenase (LDH) using a FUJI DRI-CHEM NX500V at the Chiral Material Core Facility Center of Sungkyunkwan University. Whole blood and BALF cell counts were performed using an auto hematology analyzer (Mindray, BC 5000 Vet, China) at the Chiral Material Core Facility Center of Sungkyunkwan University.

### Statistical analysis

All experiments were independently performed at least three times. Statistically significant differences were determined using the unpaired t-test. Graphpad Prism software was used for statistical analyses. Data are reported as mean ± SEM with significance set at *P* < 0.05. P-values and detailed information for each experiment are provided in the figure legends.

## Results

### Synthesis and characterization of lung-homing nanoliposome

**Fig. 1:**
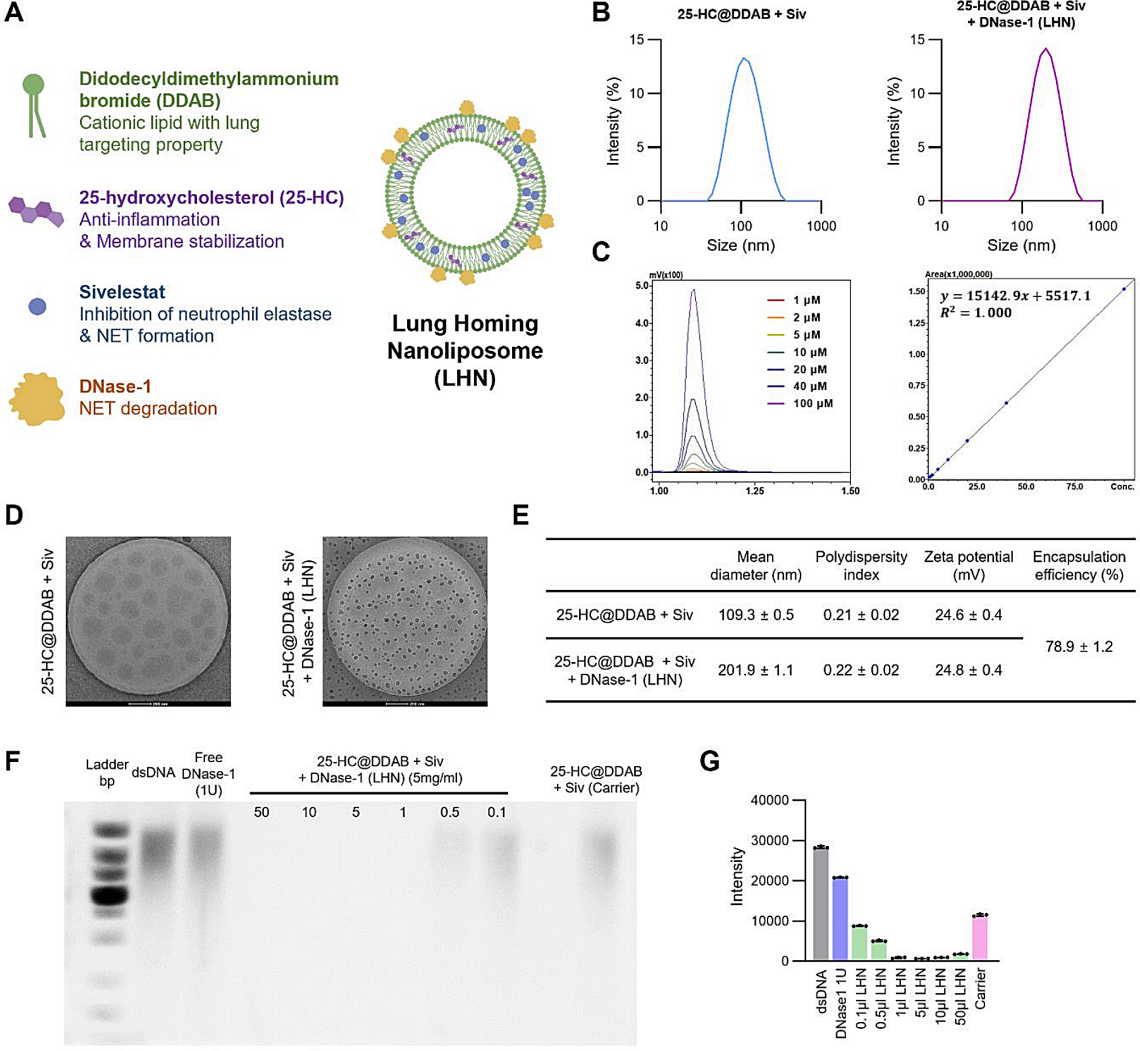
Physicochemical characterization of LHN. (**A**) Composition of LHN. (**B**) Size distribution of 25-HC@DDAB + sivelestat or 25-HC@DDAB + sivelestat + DNase-1. (**C**) Encapsulation efficiency of 25-HC@DDAB + sivelestat + DNase-1 (**D**) Cryo-electron microscopy (Cryo-EM) image of 25-HC@DDAB + sivelestat and 25-HC@DDAB + sivelestat + DNase-1 (LHN). Scale bar, 200 nm. (**E**) Characterization of LHN. Intensity averaged hydrodynamic diameters, PDI, zeta potential values and encapsulation efficiency are presented. (**F**) DNase-1 activity assay showing the migration profile of pure DNA after digestion with free DNase-1, LHN, and 25-HC@DDAB + sivelestat, along with varying concentrations of LHN (**G**) Quantification of DNase-1 activity assay intensity

In this study, we formulated LHN by incorporating a lung-targeting moiety by using a combination of cationic lipid (DDAB) and 25-hydroxycholesterol into the nanoliposome structure we demonstrated in our previous research [25]. To promote NETosis inhibition and therapeutic efficacy we included sivelestat, a NE inhibitor, within the formulation. Additionally, DNase-1 was coated onto the nanoliposomes to facilitate the degradation of NETs that are released both prior to and beyond the administration of the nanoliposome (Fig. 1A). The lung-homing nanoliposomes (LHNs) were characterized by dynamic light scattering (DLS) and laser doppler electrophoresis analysis, measuring their mean diameter, polydispersity index (PDI), and zeta potential. Dynamic light scattering analysis indicated that uncoated LHNs had a mean diameter of 109.3 ± 0.5 nm. After the DNase-1 coating process, the mean diameter increased to 201.9 ± 1.1 nm, indicating successful conjugation of DNase-1 onto the nanoliposomes (Fig. 1B). Additionally, the nanoliposomes maintained a positive zeta potential, as the inclusion of DNase-1 did not significantly affect the zeta potential, which remained at + 24.6 ± 0.4 mV and + 24.8 ± 0.4 mV respectively for each formulation (Fig. 1E). All formulations exhibited a polydispersity index (PDI) of approximately 0.2, indicating a homogeneous population of spherical nanoliposomes confirmed by cryo-electron microscopy (Cryo-EM) images (Fig. 1D and E).

In addition, LHNs were characterized by the encapsulation efficiency of sivelestat and activity of the coated DNase-1. Encapsulation efficiency (EE) of sivelestat in LHN was determined by disrupting the nanoliposomes with acetonitrile (dilution 1:100) and analyzing the samples using high-performance liquid chromatography (HPLC). HPLC results showed that modified concentration of sivelestat from 1 to 100 µM maintained a high encapsulation efficiency, achieving an encapsulation efficiency of 78.9 ± 1.2% in the optimal composition (Fig. 1C). To confirm the enzymatic activity of DNase-1 immobilized on the LHN surface, DNA degradation assays were conducted (Fig. 1F and G). The nanoparticle-bound, long-acting DNase-1 effectively degraded DNA at concentrations exceeding 1ul (5 mg/mL).

### Lung-homing nanoliposome suppresses NETosis factors and SIRS

**Fig. 2:**
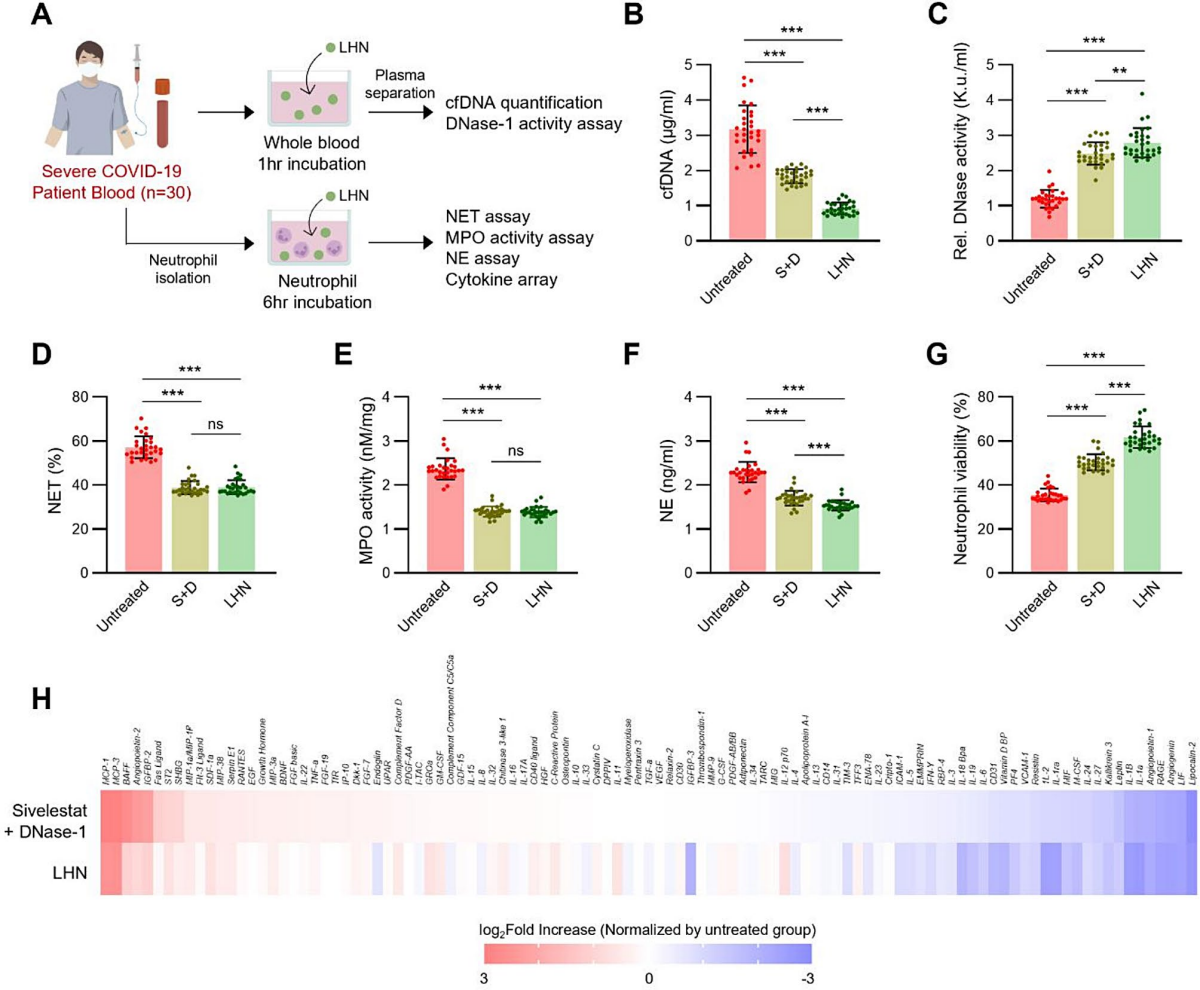
LHN suppressed NETosis factors and SIRS. (**A**) Schematic illustration of assays assessing LHN-mediated inhibition of NETosis in blood samples from severe COVID-19 patients. (**B**-**H**) Plasma and neutrophil samples were collected from severe COVID-19 patients. (*n* = 30) (**B**) cfDNA levels and (**C**) relative DNase-1 activity in the plasma. (**D**) NET, (**E**) MPO, (**F**) NE and (**H**) Cytokine levels in neutrophils are isolated from these patients. (**G**) Neutrophil viability was evaluated 6 h post-LHL treatment

Regulating NETosis in the early stages of infection is crucial for preventing the detrimental effects of excessive inflammation and neutrophil activation. Excessive NETs prolong inflammation and cause lung tissue damage, leading to impaired lung function and fibrosis. In our previous research, we found that severe septic COVID-19 patients hospitalized with poor prognosis exhibited excessive NETosis, as evidenced by elevated levels of cell-free DNA (cfDNA), NET, heightened myeloperoxidase (MPO) activity, and increased NEs. This excessive NETosis led to depleted DNase activity due to the uncontrollable formation of NETs [19]. To assess the inhibitory effects of LHN on NETosis and cytokine production, we collected fresh blood samples from patients with SARS-CoV-2 infection (*n* = 30) (Fig. 2A), who were admitted to the intensive care unit (ICU), exposed at a higher risk of severe ARDS. We treated the plasma of COVID-19 patients with either free sivelestat and DNase-1 or LHNs. Consistent with our previous findings, patients exhibited elevated plasma levels of cfDNA, a recognized biomarker indicative of NET formation. Treatment with LHN resulted in a significant reduction of cfDNA concentrations (Fig. 2B), indicating decreased formation and degradation of NETs. Concurrently, relative DNase-1 activity in the plasma was substantially increased following treatment of exogenous DNase-1 by LHN treatment (Fig. 2C), suggesting enhanced degradation of NET components.

Neutrophils isolated from these patients exhibited exaggerated levels of NETosis activity as LHN treatment markedly reduced NET formation in these neutrophils (Fig. 2D). Furthermore, the levels of myeloperoxidase (MPO) and NE, key granular enzymes involved in NETosis, were significantly decreased in LHN treated neutrophils (Fig. 2E and F). Importantly, LHN treatment resulted in higher neutrophil viability at 6 h post-treatment (Fig. 2G), indicating that the inhibition of neutrophil activation by LHN led to a reduction in NETosis-induced cell death.

Subsequent to these findings, cytokine array analyses of plasma from COVID-19 patients demonstrated a reduction in pro-inflammatory cytokine levels (Fig. 2H). Excessive production of pro-inflammatory cytokines such as interleukin-1β (IL-1β), interleukin-6 (IL-6), interleukin-8 (IL-8), and interleukin-17 A (IL-17 A) alongside other proinflammatory cytokines by neutrophils is known to play a pivotal role in the formation of NETs and the initiation of SIRS [26–29]. These cytokine reductions may inhibit neutrophil priming, activation, and subsequent infiltration into pulmonary tissue. Notably, LHN treatment resulted in a significantly greater reduction of these cytokines than treatment with free sivelestat and DNase-1 (Fig. 2H). These data suggest that LHN effectively suppresses NETosis by inhibiting neutrophil activation and enhancing the degradation of released NETs, thereby potentially attenuating SIRS in COVID-19 patients.

### LHN enhances survival and mitigates pulmonary tissue damage in a fibroproliferative ALI model

**Fig. 3:**
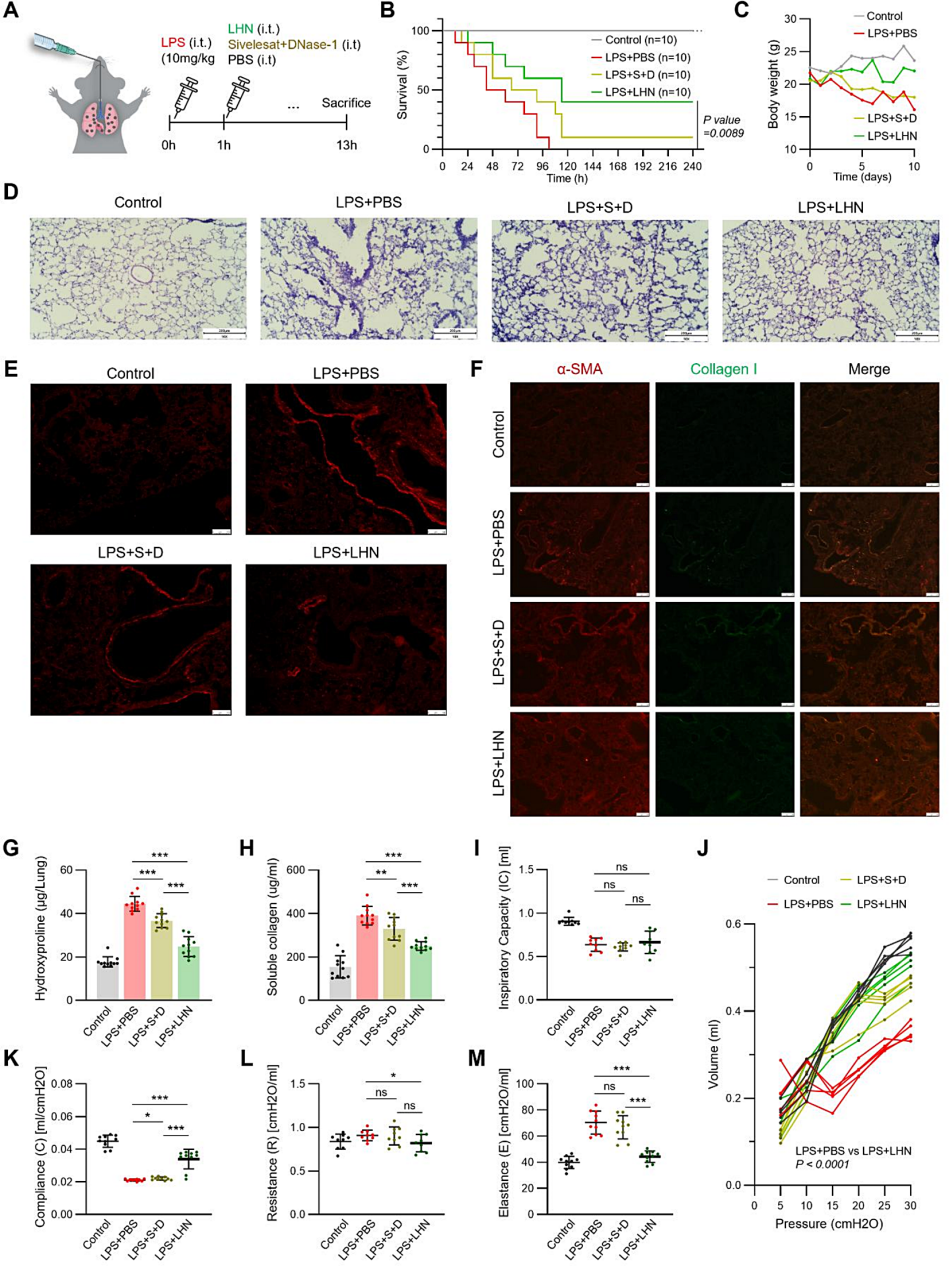
LHN improved mortality and mitigated lung tissue damage and lung function in fibroproliferative ALI (**A**) Schematic representation of the ALI model development. ICR mice were administered LPS (10 mg/kg) via intratracheal (i.t.) injection to induce ALI. One hour later, LHN, sivelestat and DNase-1, or PBS were injected intratracheally. Mice were sacrificed 13 h post-injection for further analysis. (**B**-**C**) ALI model in mice after administration of sivelestat and DNase-1 and LHN (*n* = 10 per group). (**B**) Time-course of survival rate. (**C**) Time-course of body weight. (**D**) Hematoxylin and eosin (H&E) analysis of lung tissue from ALI mice models. Representative images from each group are shown (*n* = 5). Scale bar, 200 μm. (**E**) TUNEL assay of lung tissue from ALI mice models. Representative images from each group are shown (*n* = 5). Scale bar, 100 μm. (**F**) Immunohistochemistry for α-smooth muscle actin and collagen I in lung tissue from ALI mice models. Representative images from each group are shown (*n* = 5). Scale bar, 100 μm. (**G**-**H**) Effect of LHN on (**G**) Lung Tissue Hydroxyproline Content and (**H**) BALF Collagen Levels in ALI Mice (**I**-**M**) Respiratory Function is indicated by (**I**) inspiratory capacity (IC), (**J**) PV curve, (**K**) lung compliance, (**L**) resistance, and (**M**) elastance. *P < 0.05, **P < 0.01 and ***P < 0.001. A Student unpaired, two-tailed t test was used for comparison of between-group data

Building upon our findings that LHN treatment suppresses NETosis and reduces cytokine production in COVID-19 patients derived neutrophils, we attested its therapeutic potential by investigation in mouse models. Regulating neutrophil activation and proinflammatory response during the early stages of lung injury may enhance pulmonary function and improve overall survival rates. Therefore, to test the efficacy of LHN, we selected LPS-induced ALI model which is well-established for inducing pronounced neutrophilic alveolitis and increased lung permeability. The ALI model shares the same pathophysiological progression as ARDS, but a milder pathology. We used the term acute lung injury because we could not observe the severe pathology associated with ARDS at the acute phase of the disease progression. For the ALI model, mice were administered with a high dose of LPS (10 mg/kg) via intratracheal injection, followed by intratracheal administration of LHN, a combination of sivelestat and DNase-1, or PBS (Fig. 3A). Survival analysis demonstrated that at 10 days post-treatment, LHN administration significantly improved survival rates to 40%, compared to 0% survival in the LPS + PBS group and 10% survival in the group receiving the free drug, demonstrating a markedly improved prognosis (Fig. 3B). Additionally, LHN treated mice exhibited less body weight loss throughout the experiment (Fig. 3C), suggesting reduced disease severity.

**Fig. 4:**
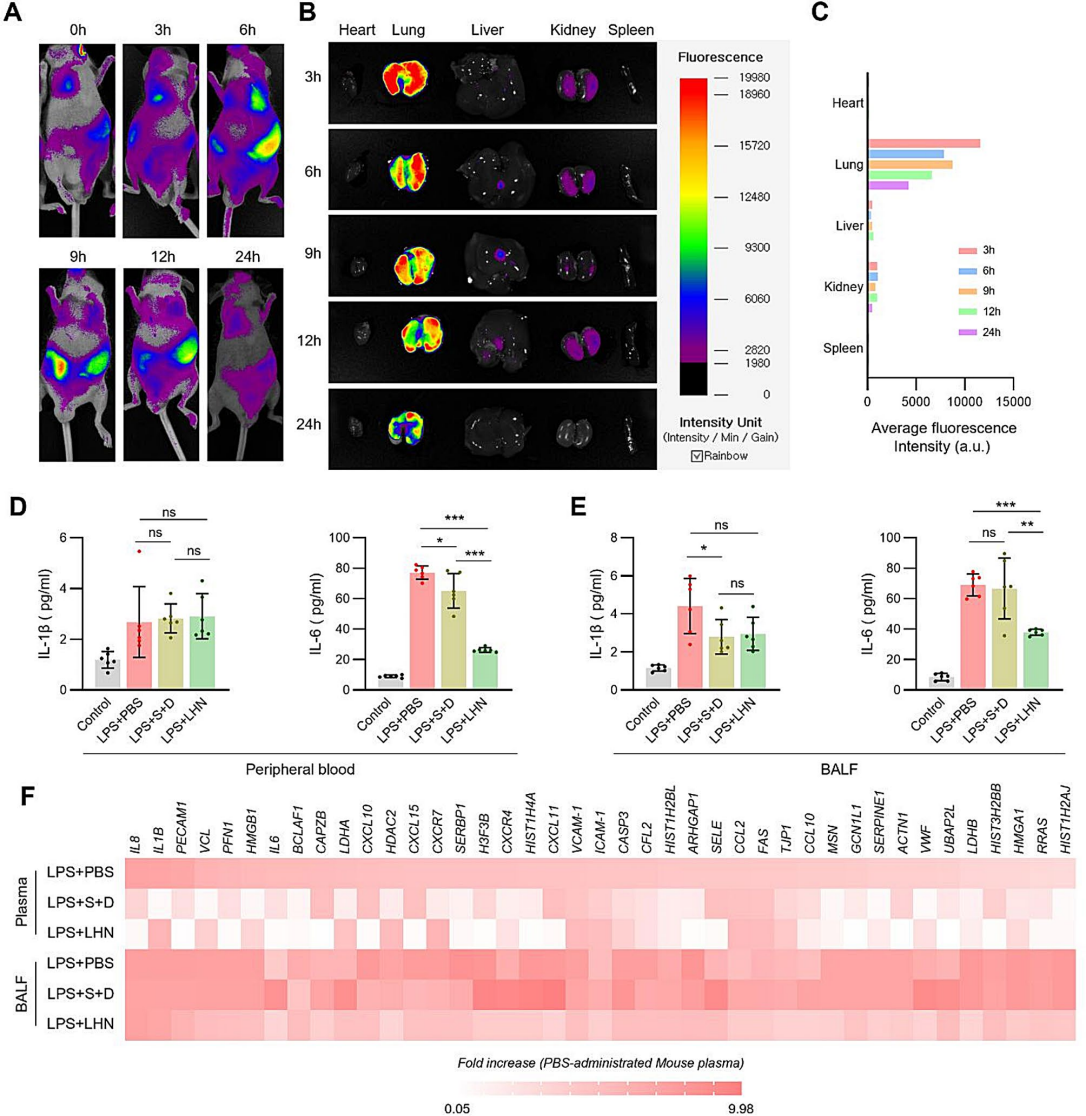
Inflammation caused by ALI is suppressed by lung-homing nanoliposomes resident within the lung space (**A**-**C**) BALB/C nude mice were administered LPS (10 mg/kg) via intratracheal (i.t.) injection, followed by the intratracheal injection of Cy5.5-labeled LHN. Fluorescence expressions were monitored to evaluate biodistribution and lung-targeting efficiency. (**A**) In vivo imaging of LHN at 0 h, 3 h, 6 h, and 12 h after intratracheal administration of Cy5.5-labeled LHN. (**B**) Ex vivo imaging of dissected organs at 12 h and 24 h post-administration of Cy5.5-labeled LHN (**C**) Quantification of the average fluorescence intensity from ex vivo imaging at 12 h and 24 h. (**D**-**F**) ALI was induced in ICR mice via intratracheal (i.t.) injection of LPS. After establishing the ALI model, mice were treated with either LHN or a combination of sivelestat and DNase-1. Cytokine levels in peripheral blood and BALF were measured to assess the inflammatory response. (**D**) Quantification of IL-1β and IL-6 levels in peripheral blood. (**E**) Quantification of IL-1β and IL-6 levels in BALF. (**F**) Effect of LHN and the combination of sivelestat and DNase-1 in suppressing SIRS, as confirmed by cytokine array analysis in both BALF and plasma. (*n* = 3/each group)

Histopathological examination of lung tissues revealed that LHN mitigated pulmonary tissue damage induced by LPS. Hematoxylin and eosin (H&E) staining demonstrated that lipopolysaccharide (LPS) administration induced significant pathological alterations, including inflammatory cell infiltration, alveolar wall collapse, and interstitial as well as intra-alveolar edema. Treatment with LHN alleviated these changes in immune response and lung histopathology (Fig. 3D). Aligned with these findings, cytokine profiling revealed alterations in PECAM-1 expression, suggesting enhanced endothelial structural integrity associated with reduced immune cell permeability. Additionally, a substantial reduction in chemokines such as CXCL10, CXCL11, and CXCL15, along with lower levels of proinflammatory cytokines such as IL-1β, IL-6, IL-8, and HMGB1, mitigate neutrophil transmigration and recruitment (Fig. 4F). This modulation resulted in decreased neutrophil proportion in BALF, as further supported by complete blood cell analysis (Fig. S3F). In terminal deoxynucleotidyl transferase-mediated dUTP nick-end labeling (TUNEL) assays, following LHN treatment, apoptosis of lung epithelial cells was significantly diminished compared to the LPS and free drug-treated groups (Fig. 3E).

Furthermore, immunohistochemical analysis was conducted to demonstrate the expression of α-smooth muscle actin (α-SMA) and collagen I, in order to evaluate the initial fibroproliferative response in the damaged lungs of ALI mice (Fig. 3F). Although α-SMA, a marker of activated fibrogenic cells such as myofibroblasts, was comparably elevated across all LPS-treated groups, the synthesis of collagen I was significantly reduced when LHN was treated. Additionally, the LHN-treated group exhibited a significant reduction in hydroxyproline levels in lung tissue and collagen release in BALF compared to the LPS group, indicating attenuation of collagen deposition and extracellular matrix remodeling associated with lung injury (Fig. 3G and H). These findings suggest that LHN not only improved lung histopathological outcomes but also attenuated initial lung tissue damage and lung fibrosis progression in fibroproliferative ALI model.

### LHN alleviates respiratory function impairment induced by ALI

To investigate the impact of LHN on initial respiratory function, we performed a forced oscillation technique (FOT) using the flexiVent FX-2 system to assess the biophysical properties of pulmonary mechanics on ALI mice treated with LHN or controls. Mechanical ventilation was performed on mice via tracheostomy and several forced oscillation tests to determine various respiratory mechanics parameters (Fig. 3I and M). Pressure-volume (P–V) curve analysis indicates that LHN treatment improved total lung capacity compared to the group treated with LPS alone. However, no significant difference was observed in inspiratory capacity (Fig. 3I and J). LHN-treated mice exhibited significant improvements in lung resistance, compliance and elastance compared to the group treated with LPS alone. (Figure 3K and M). Collectively, these findings indicate that LHN effectively alleviates respiratory dysfunction associated with ALI, with predominant improvements observed in the mechanical properties of the alveoli and lung parenchyma. Assessments of the mechanical properties of the respiratory system suggest that administration of LHN leads to significant improvement of impaired pulmonary function during the initial phase of pulmonary infection.

### LHN suppresses inflammation by persistently residing within pulmonary tissue

To assess the biodistribution and lung-targeting efficiency of LHN, we administered Cy5.5-labeled LHN intratracheally to BALB/c nude mice. In vivo fluorescence imaging revealed that LHN was predominantly localized in the lung tissue up to 24 h post-administration (Fig. 4A). The concentrated fluorescence signals in the lungs indicate that the lung-targeting functionality of the 25-HC@DDAB structure remained intact effectively following the incorporation of sivelestat and DNase-1. At six hours post-injection in mice, parts of fluorescent signals were detected caudal to the pulmonary region, which likely represents nanoliposomes present in the stomach. These nanoliposomes were likely transported through mucus and periciliary liquid propelled by the coordinated movement of epithelial cilia toward the oropharynx, subsequently swallowed, and reabsorbed through the gastrointestinal tract. Although the majority of LHNs are localized within the lung tissue following intratracheal administration, there remains the potential for nanoliposomes to translocate into the systemic circulation via passive diffusion across the thin alveolar epithelial-endothelial barrier. Ex vivo imaging of dissected organs (heart, lung, liver kidneys and spleen) at 3 to 24 h confirmed persistent retention of LHN in the lungs, with negligible fluorescence observed in other organs (Fig. 4B and C).

In the setting of ALI model, the altered microenvironment within epithelial cell damage such as loosened endothelial and epithelial barrier integrity and pulmonary edema may influence the biodistribution of lung-homing nanoliposomes (LHNs). LPS injected mice that were administered Cy5.5-labeled LHN showed that the nanoliposomes were consistently concentrated in the lungs after 24 h (Fig. S2). Strong fluorescent signals in lung tissue can imply lung targeting moiety of positively charged DDAB and 25-HC liposome structure, attributed by transient aggregation with red blood cells (RBCs) shown in previous reports [25]. Moreover, there was no organ damage that could be caused by systemic blood circulation, which was evidenced by GOT/GPT (liver damage) (Fig. S4A-S4B), BUN/creatinine (kidney damage) (Fig. S4C-S4D) and LDH (total organ damage) (Fig. S4E). Based on the CBC analysis results, there was no adverse effect on the distribution or number of blood cells in peripheral blood (Fig. S3A-S3D).

In the ALI model, lung resident of LHN led to a significant reduction in pro-inflammatory cytokines in both peripheral blood and BALF (Fig. 4D and F). Specifically, levels of IL-6 were markedly decreased in the blood (Fig. 4D) and BALF (Fig. 4E) of LHN-treated mice compared to LPS treated controls. Cytokine array analysis further confirmed the broad-spectrum anti-inflammatory effects of LHN, especially showing reduced expression of multiple cytokines and chemokines involved in infiltration and activation of neutrophils. (Fig. 4F). These data indicate that LHN effectively suppresses initial inflammation systematically by persistently localizing within pulmonary tissue and orchestrating the local immune response in lung tissue.

### Anti-NETosis effects of LHN in the ALI model

**Fig. 5:**
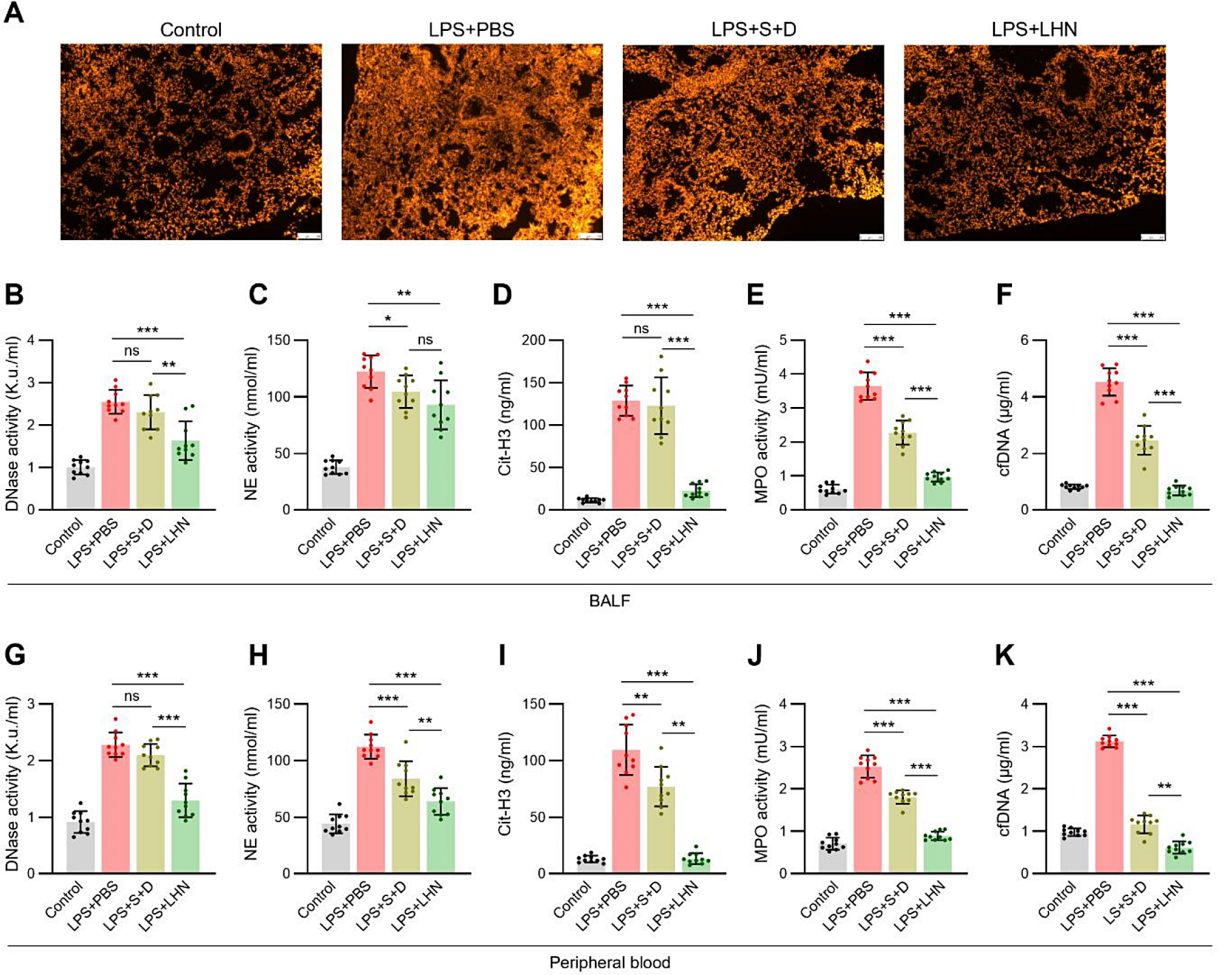
Anti-NETosis effects of lung-homing nanoliposome in the ALI model. (**A**-**K**) ALI was induced in ICR mice via intratracheal (i.t.) injection of LPS. After establishing the ALI model, mice were treated with either LHN or a combination of sivelestat and DNase-1. After 13 h post-treatment, mice were sacrificed, and BALF, blood, and lung tissues were harvested for further analysis (**A**) NETosis in lung tissue was visualized through imaging using Sytox-Orange to detect NET formation. Representative images from each group are shown (*n* = 5). Scale bar, 100 μm. (**B**) DNase activity, (**C**) NE activity, (**D**) Citrullinated Histone H3 level, (**E**) MPO activity, (**F**) cfDNA level were measured in BALF. (**G**) DNase activity, (**H**) NE activity, (**I**) Citrullinated Histone H3 level, (**J**) MPO activity, (**K**) cfDNA level were measured in peripheral blood

To further elucidate the anti-inflammatory effects of LHN, we next examined its regulative effect on NETosis in infected lung tissue. Sytox-Orange dye revealed extensive formation of NETs, characterized by extracellular DNA structures. Additionally, injured or dead lung epithelial cells resulting from overall initial pulmonary damage were featured, and colocalized among the NETs. LHN treatment markedly diminished NET formation, degradation, and associated epithelial damage in the lungs compared to LPS treated mice (Fig. 5A).

To further confirm the specific local and systemic anti-NETosis efficacy of LHN, we assessed NETosis biomarkers in both BALF and peripheral blood. Analysis in BALF sample exhibited significantly increased DNase activity (Fig. 5B) and decreased NE activity (Fig. 5C), citrullinated histone H3 levels (Fig. 5D), MPO activity (Fig. 5E), and cfDNA levels (Fig. 5F), indicating effective inhibition of NETosis. Similar trends were observed in peripheral blood samples (Fig. 5G and K), suggesting that LHN not only suppresses NETosis within the pulmonary environment but both systemically, contributing to its therapeutic efficacy in ALI. This effect is attributable to the attenuation of initial pulmonary inflammation by inhibition of NETosis within the lungs. Additionally, as suggested by the biodistribution results, LHN that entered the systemic circulation likely exerted systemic anti-NETosis effects on circulating neutrophils recruited to the pulmonary tissue, supported by a more balanced NE activity observed in peripheral blood samples with higher significance.

## Discussion

In clinical practice, the onset of ARDS in high-risk patients presents a critical emergency due to its rapid progression toward severe pulmonary dysfunction and SIRS. This can induce a hyperinflammatory cascade affecting multiple tissues and organs, frequently resulting in MOF and high risk of mortality. Moreover, the release and sustained presence of NETs has a pivotal role in the pathogenesis of ARDS. Although sivelestat is well-established for reducing pulmonary vascular permeability, suppressing airway mucus secretion, while lowering pro-inflammatory cytokine production, some studies have shown mixed outcomes on long-term survival rates [22, 30, 31]. The limited efficacy may be attributed to the short half-life (2–3 h) and lack of targeted lung delivery, thereby diminishing therapeutic impact. Yet, dosage escalation in high-risk patients must be approached with caution, as an overdose of NET inhibitors could compromise immune function, heightening vulnerability to additional infections [21]. Additionally, while recombinant human DNase-1 effectively degrades NET-associated DNA, there were limitations in application by a notably short plasma half-life (4–5 h) [32]. To address these challenges, recent studies have shown that advanced drug delivery systems enhance circulation time and therapeutic potential [19, 33].

By incorporating sivelestat and DNase-1 into the 25-HC@DDAB design, this approach aims to selectively target lung tissue, providing prolonged bioavailability while inhibiting NET formation and enzymatically degrading pre-existing NETs. The multi-action mechanism of LHN offers an approach to manage neutrophil-mediated damage, thereby curbing the downstream inflammatory cascade along with the anti-inflammatory effects of 25-HC. In critically ill patients with acute organ dysfunction, detecting its impact on systemic drug exposure is challenging to quantify and may result in therapeutic failures or unintended toxicities. We selected the intratracheal administration route to enable rapid, localized delivery at the early immune phase, directly reaching the lungs. This prevents risks of overdose usage in systemic administration and also diminished drug delivery to the lungs by damaged pulmonary endothelium integrity in type I ARDS. Together, these strategies underscore LHN’s potential as a first-line therapeutic intervention in ARDS, regulating both the direct effects of NETosis and broader inflammatory response by efficient lung targeting.

LHN treatment in severe COVID-19 patient-derived samples exhibited a marked reduction in NET-associated markers, such as cfDNA, MPO and NE activity. Additionally, the reduction in pro-inflammatory cytokines like IL-6 and IL-8 suggests that LHN not only modulates neutrophil activity but also impacts the broader inflammatory milieu. IL-8, in particular, plays a pivotal role in recruiting and activating neutrophils, and elevated levels have been associated with worse outcomes in ARDS patients [28, 34]. By reducing IL-8 levels, LHN may disrupt the positive feedback loop that perpetuates neutrophil recruitment and activation, further contributing to the resolution of inflammation.

The therapeutic efficacy of LHN was further validated in a murine model of LPS-induced ALI, supporting the therapeutic potential of LHN in vivo. LHN significantly enhanced survival rates, attenuated pulmonary tissue injury, and diminished histological evidence of fibrosis, as decreased levels of collagen I (Fig. 3A and F). In addition to the direct effects elicited by intratracheal administration of LPS, NETs are also known as critical mediators in driving fibroproliferative response. Collagen I, specifically its metabolism indicator procollagen type I N-terminal propeptide (PINP), is increasingly recognized as an early phase marker in assessing fibrosis-related ARDS prognosis [35]. Elevated BALF and serum levels of PINP in ARDS patients are associated with worse clinical outcomes, including increased lung injury severity and mortality, due to their reflection of enhanced collagen deposition and fibroproliferative dynamics [36, 37]. In early stages of ALI, LHN administration effectively decreased soluble collagen levels present in BALF and collagen I deposition in lung tissue, demonstrating attenuation of the fibroproliferative response (Fig. 3F and H). Proinflammatory signals were upregulated with the response, as reductions in specific cytokines such as IL-6, IL-8 and HMGB1 may indicate modulation of this pathway (Fig. 4F). Unlike conventional ARDS treatments that primarily focus on inflammation suppression and restoring immediate respiratory function, LHN addresses the underlying target for severe disease progression. These findings suggest that NETosis regulation through LHN presents a promising therapeutic approach to prevent persistent fibrotic remodeling, driven by myofibroblast accumulation in response to excessive immune activation and elevated levels of profibrotic mediators. This can lead to improvement of long-term pulmonary function and clinical outcomes, addressing an unmet need in current therapeutic strategies.

In clinical practice, the absence of prompt initial administration in severe ARDS patients often results in progressive respiratory failure necessitating the initiation of mechanical ventilation. Despite adopting lung-protective ventilation strategies, including low tidal volumes and optimized positive end-expiratory pressure (PEEP), significant fluctuations in lung volume can damage alveolar-capillary interface, inducing inflammation and pulmonary edema, thereby triggering a self-perpetuating cycle of worsening lung injury [38, 39]. In our ALI model, administration of LHN demonstrated early improvement in key pulmonary function parameters, including compliance, resistance, elastance, and TLC (Fig. 3I and M). These parameters are standard clinical measures for evaluating lung function, providing precise assessments of the pulmonary physiological state and holding promise for clinical implementation of LFN. By mitigating the need for excessive mechanical ventilation, LHN offers a dual benefit of reducing both the underlying pathology and potential iatrogenic complications.

In vivo biodistribution imaging validated that LHN preferentially accumulates within lung tissue up to 24 h following intratracheal administration. Notably, nanoliposomes leaked out to the system consistently retained their targeting properties for lung tissue, leading to minimal deposition in off-target organs (Fig. 4A and C). This strategy was critical for augmented anti-inflammatory efficacy, addressing distinct aspects of neutrophil-mediated damage in lungs, with additional systemic effects (Fig. 4D and F). Also, assessment of NETosis marker in BALF and peripheral blood confirmed inhibition of excessive NETosis, verified by reduced accumulation of NETs in lung tissue sections (Fig. 5). In our ALI model, LHN administered one-hour post-LPS challenge, corresponding to an early stage of the inflammatory response. This timely intervention effectively inhibited NETosis at its initial phase. As ALI progressed upon LPS administration, the endogenous compensatory mechanism to upregulate DNase-1 expression in response to excessive NETosis became diminished. Consequently, 12 h after administration, DNase-1 activity in the LHN-treated group was lower than in the group that received LPS alone. In contrast, isolated COVID-19 patient-derived blood samples were treated with LHN and analyzed one-hour post-treatment. In this setting, the exogenously supplied DNase-1 in LHN functioned effectively, leading to higher DNase-1 activity in the treated samples. Altogether, other markers of NETosis confirmed successful inhibition of NET with significant reduction in proinflammatory cytokine production. Through the evaluation of therapeutic efficacy achieved via nanoliposome-based targeted delivery, we verified that encapsulating sivelstat and DNase-1 within LHN formulation significantly extended their half-life, thereby enabling effective outcomes at than free drug administration while minimizing systemic off-target effects.

Our findings underscore the critical importance of early intervention in modulating neutrophil activity to prevent progression of severe ARDS. Despite the beneficial aspects highlighted in our research using LHN, targeting NETosis can act as a double-edged sword due to its role in pathogen removal and tissue repair. Some researches have revealed that adequate NETs after acute phase degrade cytokines and chemokines to halt neutrophil recruitment and aid in resolving inflammation [40, 41]. However, in cases of ARDS among high-risk patients in high age (over 65 years) or with comorbidities, regeneration ability of the lung epithelium architecture is often impaired and recovery does not proceed as effectively as in healthy individuals. Therefore, minimizing initial tissue damage by inhibiting excessive NETosis-associated injury and cytokine release syndrome is more imperative. In our ALI model, we successfully determined the effective dosage of LHN necessary to inhibit NETosis, thereby reducing additional pulmonary damage and bringing better prognosis. LHN utilizes clinically validated therapeutic agents, reformulated into a nanoliposome platform to facilitate efficacy and targeted delivery. This approach advances a clear translational potential with a high likelihood of clinical applicability. For clinical applications, optimal dosing regimen of LHN and employing complement concomitant pharmacokinetics in clinical use need to be elucidated to ensure maximum efficacy and safety.

## Conclusion

To address the therapeutic needs of high-risk patients with ALI, we developed LHN designed to specifically target neutrophil-mediated pathology during the acute phase of lung injury. The LHN is engineered for aerosolized delivery directly to the airways and alveoli, enhancing local drug concentration while minimizing systemic exposure. Our findings demonstrate that LHN effectively modulates pathological pulmonary processes by attenuating NET formation, suppressing pro-inflammatory cytokine production, and enhancing pulmonary function in both patient-derived samples and a murine model of LPS-induced ALI. These results underscore the therapeutic potential of LHN as a targeted intervention for initial ARDS, effectively addressing both localized lung damage and the broader systemic inflammatory response thereby offering a promising strategy to improve clinical outcomes in ALI.

## Electronic supplementary material

Below is the link to the electronic supplementary material.

Supplementary Material 1

## Data Availability

All data generated or analysed during this study are included in this published article and its supplementary information files.
