# Supplementary material for: Lung-homing nanoliposomes for early intervention in NETosis and inflammation during acute lung injury
Source: Nano Converg. 2025 Feb 3;12:8. doi: 10.1186/s40580-025-00475-4 (PMC11788270; doi:10.1186/s40580-025-00475-4)
Supplement: Supplementary file 1 — Supplementary Material 1 [file 40580_2025_475_MOESM1_ESM.docx]

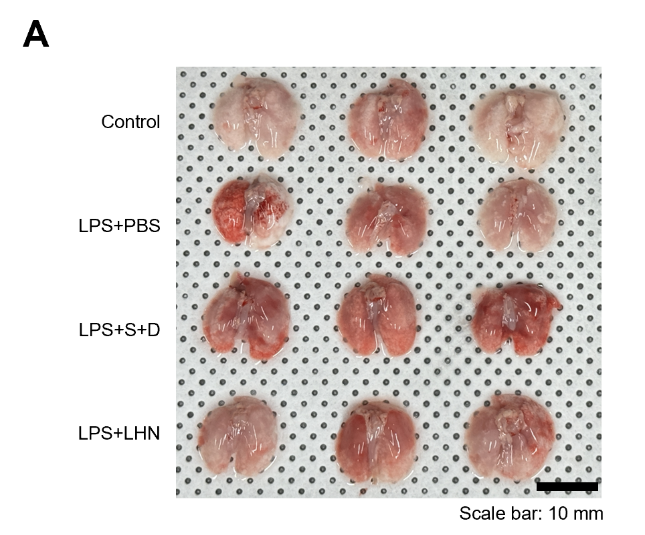


**Supplementary Fig 1. Examination of lung morphology and lesions in ALI model following administration of LHN and combination of sivelestat and DNase-1.** (A) Observation of lung morphology in the ALI model, highlighting red-colored hemorrhagic lesions and congestion in lung tissue after LPS administration, indicating inflammation and vascular damage. The effects of subsequent administration of LHN and sivelestat + DNase-1 on these pathological features are shown.


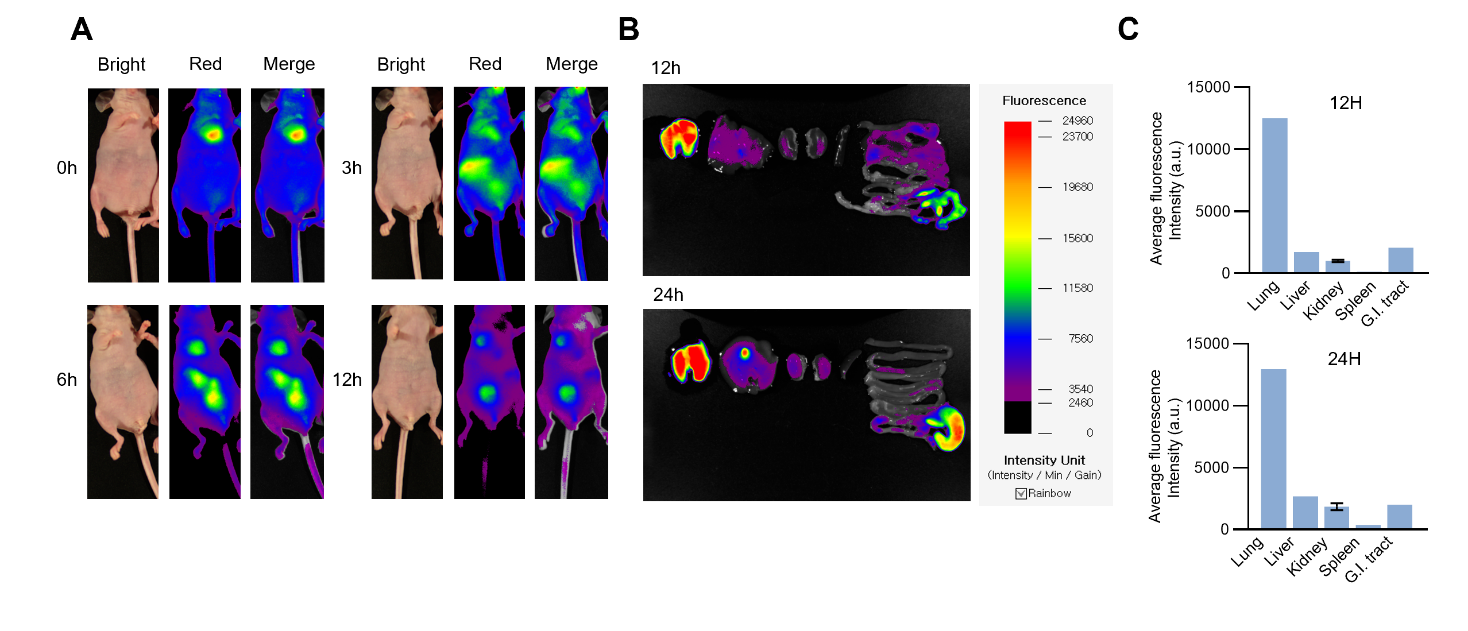


**Supplementary Fig 2. Examination of invivo biodistribution of LHN in ALI model during initial phase** BALB/C nude mice were administered LPS (10 mg/kg) via intratracheal (i.t.) injection, followed by the intratracheal injection of Cy5.5-labeled LHN. (A) In vivo imaging of LHN at 0 h, 3 h, 6 h, and 12 h after intratracheal administration of Cy5.5-labeled LHN. (B) Ex vivo imaging of dissected organs at 12 h and 24 h post-administration of Cy5.5-labeled LHN (C) Quantification of the average fluorescence intensity from ex vivo imaging at 12 h and 24 h.


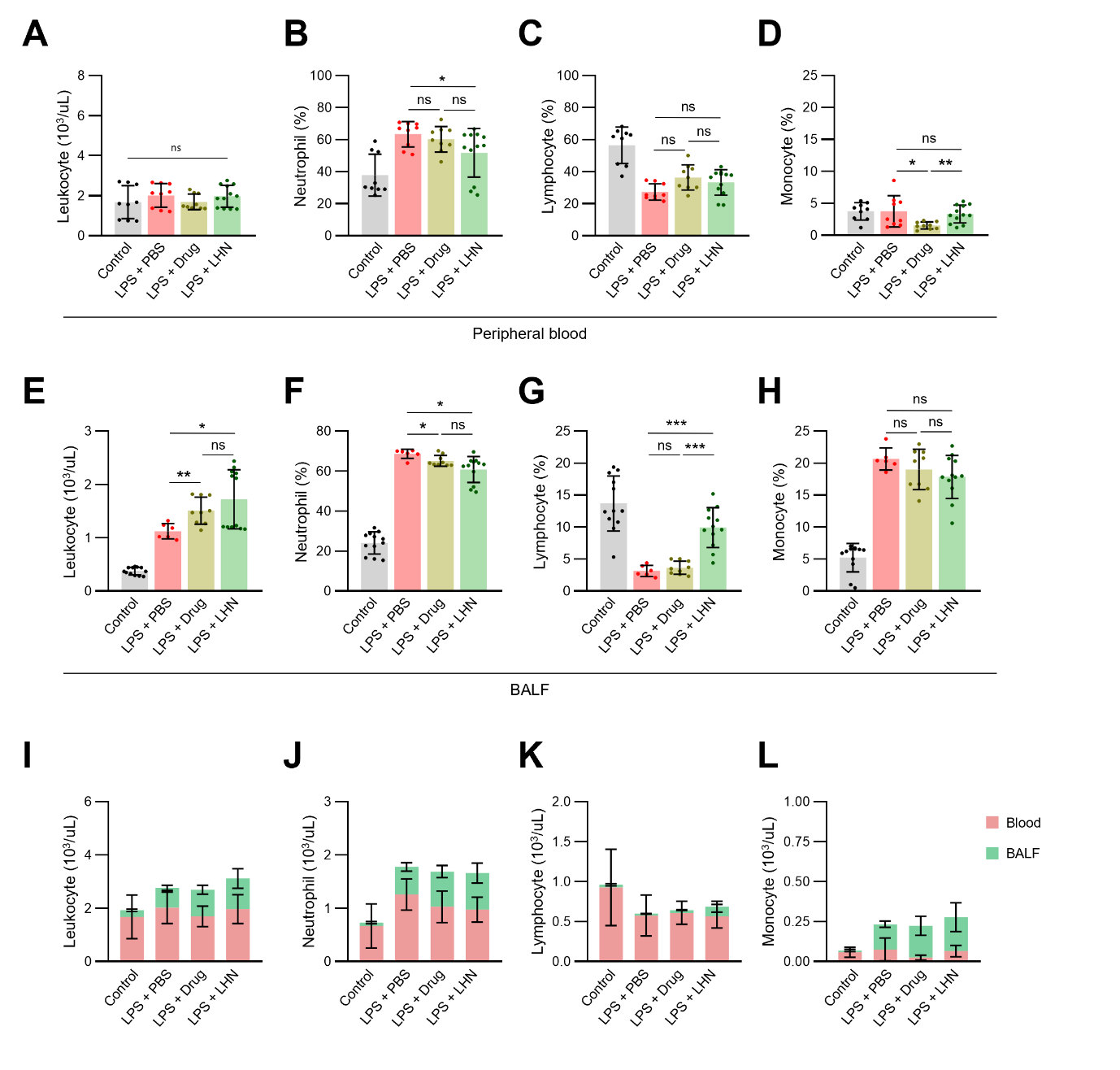


**Supplementary Fig 3. Quantified mean leukocyte counts in peripheral blood and BALF of acute lung injury mice.** Cell counts in (A-D) peripheral blood and (E-H) BALF of the acute lung injury model. (I-L) Quantified total cell counts of peripheral blood and BALF. Red bars represent blood samples, and green bars represent BALF samples. Cell counts (cells x 10^3^/μL) or cell percentages are presented as (A, E) Leukocyte (B, F) Neutrophil, (C, G) Lymphocyte, (D, H) Monocyte (n=9 each group). Statistical analysis was performed using a two-tailed unpaired t-test. ^*^ p < 0.05, ^**^ p < 0.01.


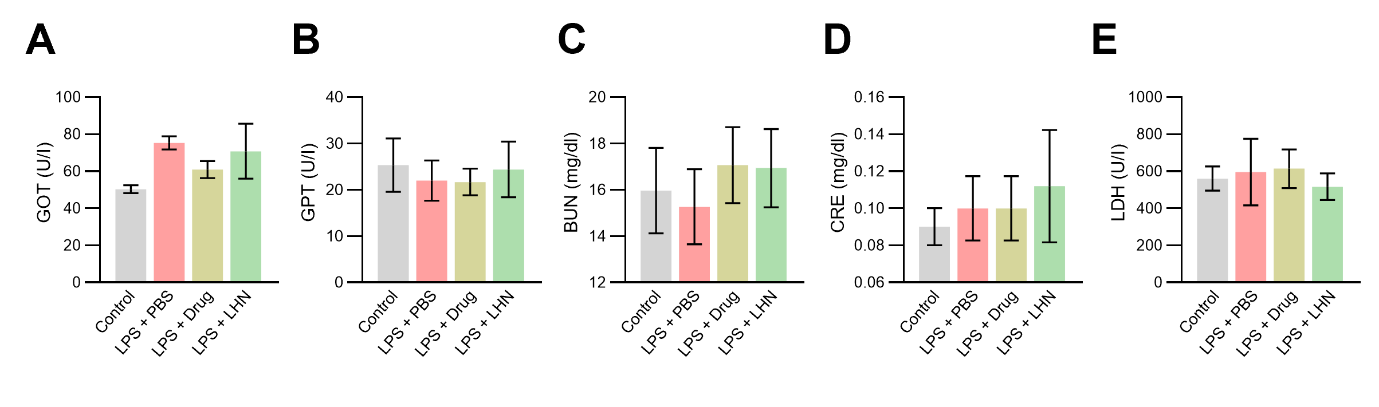


**Supplementary Fig 4. Laboratory tests in acute lung injury mice as markers for tissue damage.** (A) Glutamic oxaloacetic transaminase (GOT) (B) Glutamic pyruvic transaminase (GPT), (C) Blood urea nitrogen (BUN), (D) Creatinine (CRE), (E) Lactate dehydrogenase (LDH). Statistical analysis was performed using a two-tailed unpaired t-test. ^*^ p < 0.05, ^**^ p < 0.01.
